# The relationship between non-motor features and weight-loss in the premanifest stage of Huntington’s disease

**DOI:** 10.1371/journal.pone.0253817

**Published:** 2021-07-01

**Authors:** Wasiq Khan, Sundus Alusi, Hissam Tawfik, Abir Hussain

**Affiliations:** 1 School of Computer Science and Mathematics, Liverpool John Moores University, Liverpool, United Kingdom; 2 Department of Neurology, The Walton Centre NHS Foundation Trust, Liverpool, United Kingdom; 3 School of Built Environment, Engineering and Computing, Leeds Beckett University, Leeds, United Kingdom; Vellore Institute of Technology: VIT University, INDIA

## Abstract

Weight-loss is an integral part of Huntington’s disease (HD) that can start before the onset of motor symptoms. Investigating the underlying pathological processes may help in the understanding of this devastating disease as well as contribute to its management. However, the complex behavior and associations of multiple biological factors is impractical to be interpreted by the conventional statistics or human experts. For the first time, we combine a clinical dataset, expert knowledge and machine intelligence to model the multi-dimensional associations between the potentially relevant factors and weight-loss activity in HD, specifically at the premanifest stage. The HD dataset is standardized and transformed into required knowledge base with the help of clinical HD experts, which is then processed by the class rule mining and self-organising maps to identify the significant associations. Statistical results and experts’ report indicate a strong association between severe weight-loss in HD at the premanifest stage and measures of certain cognitive, psychiatric functional ability factors. These results suggest that the mechanism underlying weight-loss in HD is, at least partly related to dysfunction of certain areas of the brain, a finding that may have not been apparent otherwise. These associations will aid the understanding of the pathophysiology of the disease and its progression and may in turn help in HD treatment trials.

## 1. Introduction

Huntington’s disease (HD) is a devastating hereditary neurodegenerative disorder that results in cognitive and neuropsychiatric abnormalities years before the motor issues start to appear, on which the clinical diagnosis is based [1–3]. This is called the premanifest stage of the disease. Various neuroimaging studies indicated that in the premanifest-HD stage, patients show redundant brain area recruitments. Functional magnetic resonance imaging (fMRI) studies demonstrated abnormalities in cognitive domains in premanifest subjects when compared with the healthy controls in certain areas such as response inhibition [4], verbal memory [5], reward processing [6] and spatial working memory [7].

The neuropathology of HD is emphasized by degeneration of certain brain areas, specifically the striatum [8]. On a cellular level, HD is caused by a genetic defect defined by a prolonged CAG (cytosine-adenine-guanine) sequence (expansion) in the Huntington gene (HTT) [9]. Predictive genetic testing is used to identify individuals with the mutation before they develop HD motor symptoms. These patients are known as pre-disease or premanifest.

The ability to identify the abnormal gene before disease symptoms onset offers an opportunity to intervene at an early stage with potential treatments aimed at slowing disease progression or arresting it. Biomarkers are excellent tools for monitoring the disease progression and measuring efficacy of treatment interventions. Although CAG repeat length is known to correlate well with disease progression, the complex nature of the disease and its varied presentations and severity amongst individuals with the same CAG repeat length, calls for more biomarkers to understand disease progression in detail. Indeed, TRACK–HD study [10, 11] has already shown useful clinical and imaging outcome measures to be used reliably in the drug trials design.

Typically, HD starts when patients are in their thirties and forties. However, the age of onset spans from infancy and to the ninth decade. The affected individual suffers gradual functional decline and eventually requires intensive care and supervision, which often requires extensive medical input and consumes significant family and social resources [12].

In addition to the above mentioned cognitive, psychiatric and motor symptoms, HD is complicated by significant weight-loss. It is a gradual process and is known to have a negative impact on mortality and the morbidity of the disease [13]. It occurs despite high calorie intake, and is not associated with increased activity [14–21]. Furthermore, it has been shown that HD patients who have high body mass index at the initial set of symptoms indicate slow rate of the progression [22] which may provide useful indication for treatments.

It has long been established that unintended weight-loss is associated with HD disease despite high energy diets [15] and this applies to even premanifest stages of the disease [19]. This suggests that the weight-loss in HD is unlikely to be causally related to the increase in physical activity, for example, chorea. Indeed, in a randomized controlled prospective study 517 HD patients’ weight-loss was not found to correlate with motor scores such as chorea, dystonia or the total UHDRS motor score (the Unified Huntington’s Disease Rating Score). It was however found to be linked to CAG repeat length and the study findings implied that it is likely to result from a hyper-metabolic state [23]. This study had excluded patients who were taking neuroleptics which are associated with weight gain.

Other studies have shown that weight-loss is an integral part of the disease and could be in part related to hypothalamic atrophy and dysfunction [24]. Evidence of hypothalamic involvement by the disease pathology has been demonstrated in a number of studies [25]. It may therefore be anticipated that the onset of weight-loss is related, at least in part, to the onset of disease process, outside the striatum and cortex particularly the hypothalamus. This is particularly important when assessing therapeutic agents’ effect on disease progression. Current therapeutic trials are concentrating on treating HD in the premanifest stage or earlier. The presence of neuronal dysfunction, whilst gene carrying individuals are still functioning, normally suggests that disease-modifying interventions should be initiated during this period [10].

There exist a relatively limited range of studies that utilize the machine intelligence for multi-factor analysis and early prediction of disease. For instance, study in [26] addresses the use of pattern analysis and machine learning in the premanifest-HD subjects while using various types of imaging datasets. The study indicated that 76% classification accuracy of premanifest-HD versus the control subjects. The authors also indicated that using MRI measures as biomarkers can be a useful approach for assessing the neuroprotective therapies in neurodegenerative disease. Orrù et al. [27], investigated the use of support vector machine as method for the categorisation of patients’ unseen data into predefined groups using imaging biomarkers for psychiatric and neurological diseases. Their analysis indicated that the support vector machine and other machine learning algorithms provide promising results, however substantial theoretical and practical works need to be performed to implement the outcome in practical neurology and psychiatry. In [28], the authors indicated that there are various degrees of heterogeneity among patients holding HD gene in which neuroimaging can provide important cues to understand these heterogeneities. The authors claimed that most of the research in the area of neuroimaging in HD concentrated on the differences between healthy and HD patients. In their work, the authors indicated that using a combination of clinical and various types of symptoms with multimodal approach can distinguish between the neural substrates of various kinds of symptoms suffered by the HD patients.

Although aforementioned existing works focus on weight-loss in HD, these studies do not specifically address the multi-dimensional associations between weight-loss and non-motor factors specifically, at the important premanifest stage. Furthermore, the relationship between the weight-loss and various non-motor factors at premanifest stage is never investigated. These aspects should be investigated with appropriate statistical algorithms to analyse the complex associations of weight-loss at early stages of HD which is the main motivation of this study. Whilst large studies such as TRACK-HD [10] have identified important biological and clinical parameters that can be used in longitudinal studies, weight-loss may also prove to be an important and easily measurable surrogate marker for disease progression particularly at the early stages which in turn could be helpful in therapeutic trials.

In order to further explore weight-loss in HD, the authors have undertaken this study to model the associations between multiple factors specifically non-motor factors such as cognitive, psychiatric factors and independence levels and weight-loss in premanifest-HD and family control subjects. The pathophysiology of weight-loss in HD is far from clear and hence adopting an open-minded approach is useful in unravelling such a complex problem. Deploying advanced data analytics techniques might have an advantage over the conventional statistical methods to analyze the complex patterns and potential associations between multiple predefined factors and weight-loss. In this paper, we propose the implementation of an intelligent approach utilizing pattern matching, class rule mining and domain knowledge for the analysis of multiple factors and corresponding associations to severe weight-loss in the premanifest stage of HD. For the first time, we attempt to investigate *which specific fine-grained cognitive*, *psychiatric and functional factors might be highly associated with the severe weight-loss in premanifest-HD*?

The major contributions of this work include a) The deployment of HD experts’ clinical knowledge for data representation and weight categorisation, b) Establishing whether there is a strong association between the non-motor features and weight loss in HD?, c) Pattern analysis by modeling the complex associations using self-organising maps and class rule mining that is not possible with conventional statistical tools, d) Effective visualization of high dimensional associations within the lower space using machine intelligence, e) Interpretation of complex associations in form of distinct, significant and human understandable rules.

This manuscript is organized as follows: Section 2 describes the detailed proposed methodology of knowledge representation, pattern analysis and association mining. The experimental results and interpretation of representative rules are reported in section 3 followed by discussion of the findings in section 4 and finally, the conclusion and future directions are presented in Section 5.

## 2. Methodology

**Fig 1:**
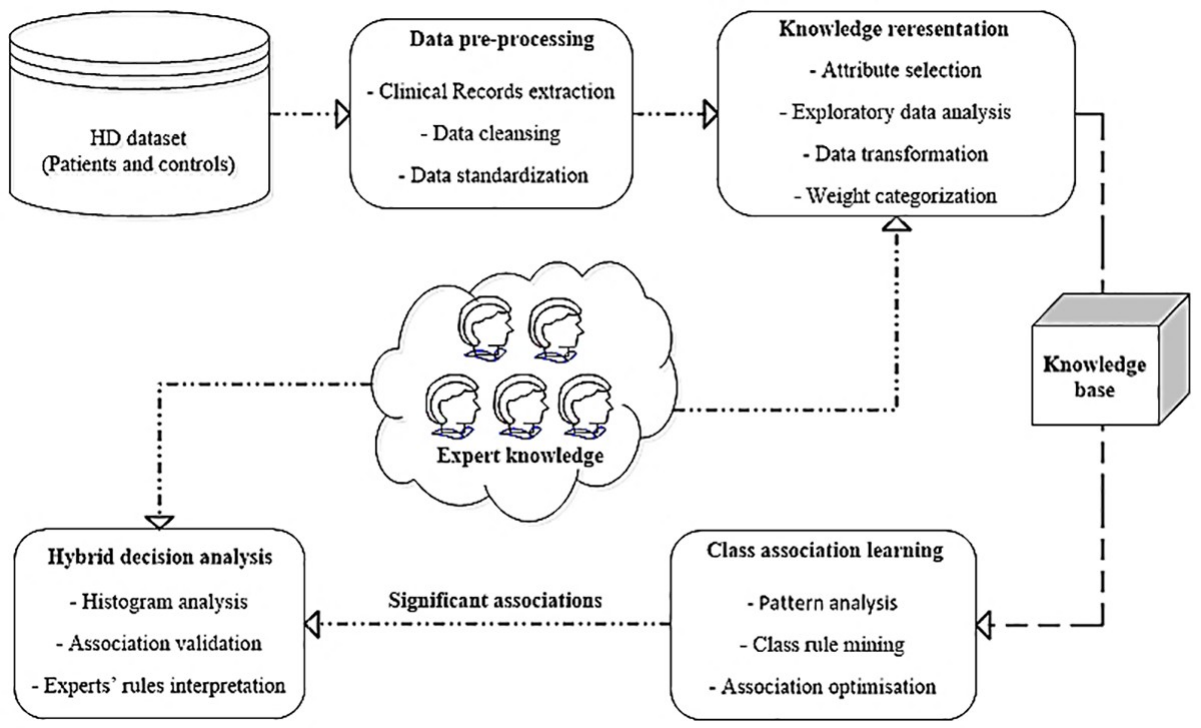
A hybrid diagnostic approach using clinical domain knowledge and data analytics to learn the association between severe weight-loss and biological factors in HD premanifest and control groups.

Composite of data analytics techniques along-with the expert knowledge are utilized in this study to analyse the complex patterns within the clinical records of HD dataset and to investigate the significant associations between weight-loss and clinical factors specifically, for the early stages of the disease. Fig 1 demonstrates the sequential procedure used in this study for the hybrid analysis of identified associations and patterns. In the first step, the HD dataset comprising patients and controlled subjects’ clinical records, is pre-processed to remove anomalies and is then transformed into categorical form using clinical experts’ knowledge. The processed dataset in the next step is then fed into different pattern analysis algorithms to extract the human understandable patterns and significant associations between desired factors. These outputs are then validated using the standard statistical measures as well as human experts to form the concluding remarks about inter-relationships between non-motor factors and weight-loss at premanifest stage of the HD. Details about dataset, pre-processing, pattern analysis and rule interpretation procedures are provided in the following sections.

### 2.1 Dataset

Enroll-HD is a global clinical research platform designed to facilitate clinical research in Huntington’s disease. The dataset used in proposed study is owned by CHDI foundation (a biomedical research organization devoted to HD) [29]. Core datasets are collected annually from all research participants as part of this multi-center longitudinal observational study. Data is monitored for quality and accuracy using a risk-based monitoring approach. All sites are required to obtain and maintain local ethical approval. We used the fourth Enroll-HD periodic dataset comprising the clinical data from 15301 participants in total (premanifest/premotor, manifest/motor-manifest HD, genotype negative and family control). Access to required HD dataset was granted to authors by the CHDI team. For further details about appropriate procedures, ethical approvals, contact details, and required terms and conditions can be found in [29]. In this study, we use the premanifest (pMan-HD) and family control (fCont-HD), records from the Enroll study comprising 8012 and 4427 records in total respectively. A fCont-HD subject is defined as a person who is living/caring for the premanifest HD subject but does not have the HD gene themselves, e.g., spouse. The dataset contains a variety of factors (i.e., variables) including the motor scores, functional abilities, cognitive, and psychiatric scores etc. As the focus of this study is to investigate the association between severe weight-loss and non-motor problems in HD, we include following factors: Cognitive scales: MMSE (mini mental state examination) and sdmt (Symbol Digit Modality Test); functional independence and capacity scores: indepscl (subjects’ independence), fiscore (functional score), tfcscore (total functional capacity score); and psychiatric assessments: exfscore (executive function), aptscore (apathy score), irascore (irritability/aggression score), depscore (depression score), sdmt (symbol digit modality test), psyscore (phsychosis score) and wtCat (weight categories). These factors are selected following the recommendations from clinical HD experts. Further explanation of these factors, detailed data capturing procedures and information about PDS4 are available in [29].

### 2.2 Data preparation and domain knowledge representation

**Fig 2:**
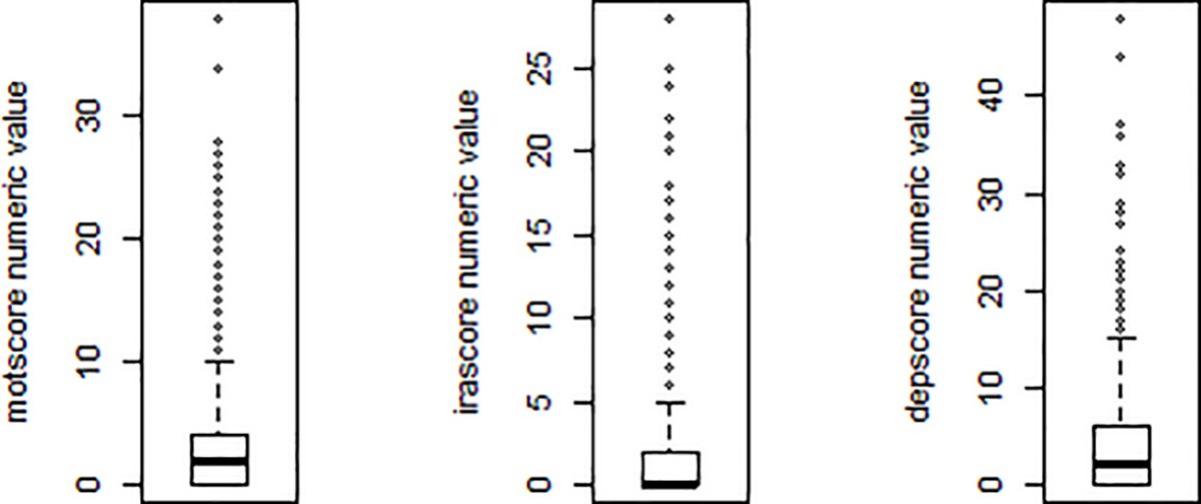
Outlier removal examples using boxplot within the motscore, irascore and depscore factors in pMan-HD records.

In the first step, we extract the required pMan-HD and fCont-HD records from the PDS4 dataset both containing the aforementioned list of selected factors. We then cleaned the datasets by eliminating the missing values/records and the outliers in each factor. More specifically, the numeric factors such as tfscore, indepscore, irascore and motscore contains outliers that were removed from both pMan-HD and fCont-HD datasets. Fig 2 shows an example of outliers within the multiple factors in pMan-HD that were eliminated using box plot. The next and important step after the data cleansing is to calculate the change in subjects’ weight over time. The dataset in the primary form contains multiple records for each participant representing the ‘*baseline’* and ‘*follow-up*’ assessments/measurements recorded annually. Most of the subjects contains 3–5 ‘*follow-up’* records excluding the baseline. As per research question, we consider the weight change (*Δw*) per subject across the entire period (i.e., from *baseline* to last *follow-up*). The *Δw* for each subject is calculated using the baseline and last follow-up records as:

$$\Delta w=\left(\frac{w_{f}-w_{b}}{w_{b}}\right)\times 100$$ (1)

Where *w*_*b*_ and *w*_*f*_ represent the baseline and last follow-up weight of a subject respectively. Algorithm 1 shows the procedure of *Δw* calculation within the pMan-HD and fCont-HD datasets. For each unique participant, the records are searched for the corresponding baseline and last follow-up sample to measure the *Δw*. The algorithm runs recursively for each participant (i.e., IDs) and corresponding follow-up records (i.e., T) with complexity of O(M * N) where M and N indicate the number of unique identities (i.e., participants) and corresponding follow-ups respectively. This leads to eliminate the baseline and all intermediate follow-up records from the datasets with remaining 1978 and 1191 data samples within the pMan-HD and fCont-HD respectively, where each data sample represents an individual’s non-repeated record.

**Algorithm 1.** Calculation of percentage weight change (*Δw*) within the pMan-HD and fCont-HD.

    **INPUT:** HD dataset D in raw form

    **OUTPUT:** Output dataset OD with weight-change (Δw) measure

    **PROCEDURE:**

        - Let **IDs** contains the list of subjects’ identifications (ID)

        **Loop A: Foreach** id in IDs

            1. Find matching records in **D** (i.e. all records for 1 subject)

            2. Store the records in a temporary data-frame **T**

            3. Loop B: Foreach record r in T

                a. **IF** r is a ‘Baseline’, **THEN**

                    i. Store the weight ***w_b_*** = weight (baseline)

                b. **IF** r is the last ‘Follow-up’, **THEN**

                    i. Store the weight ***w_f_*** = weight (last follow-up)

            **4. End (Loop B)**

            5. Calculate the delta **Δ*w*** using Eq (1)

            6. Update the weight-Change **Δ*w***[id] in OD

            7. Reset ***w_b_***, ***w_f_***, **T** to NULL

        End (Loop A)

    **RETURN: OD** (comprising Δ*w* for each subject)

Once the *Δw* is calculated and unnecessary records are filtered out from the datasets, the next step is to utilize the domain knowledge to transform the data into a Knowledge Base (KB) that will be useful for pattern analysis and identification of potential associations between the clinical factors.

**Fig 3:**
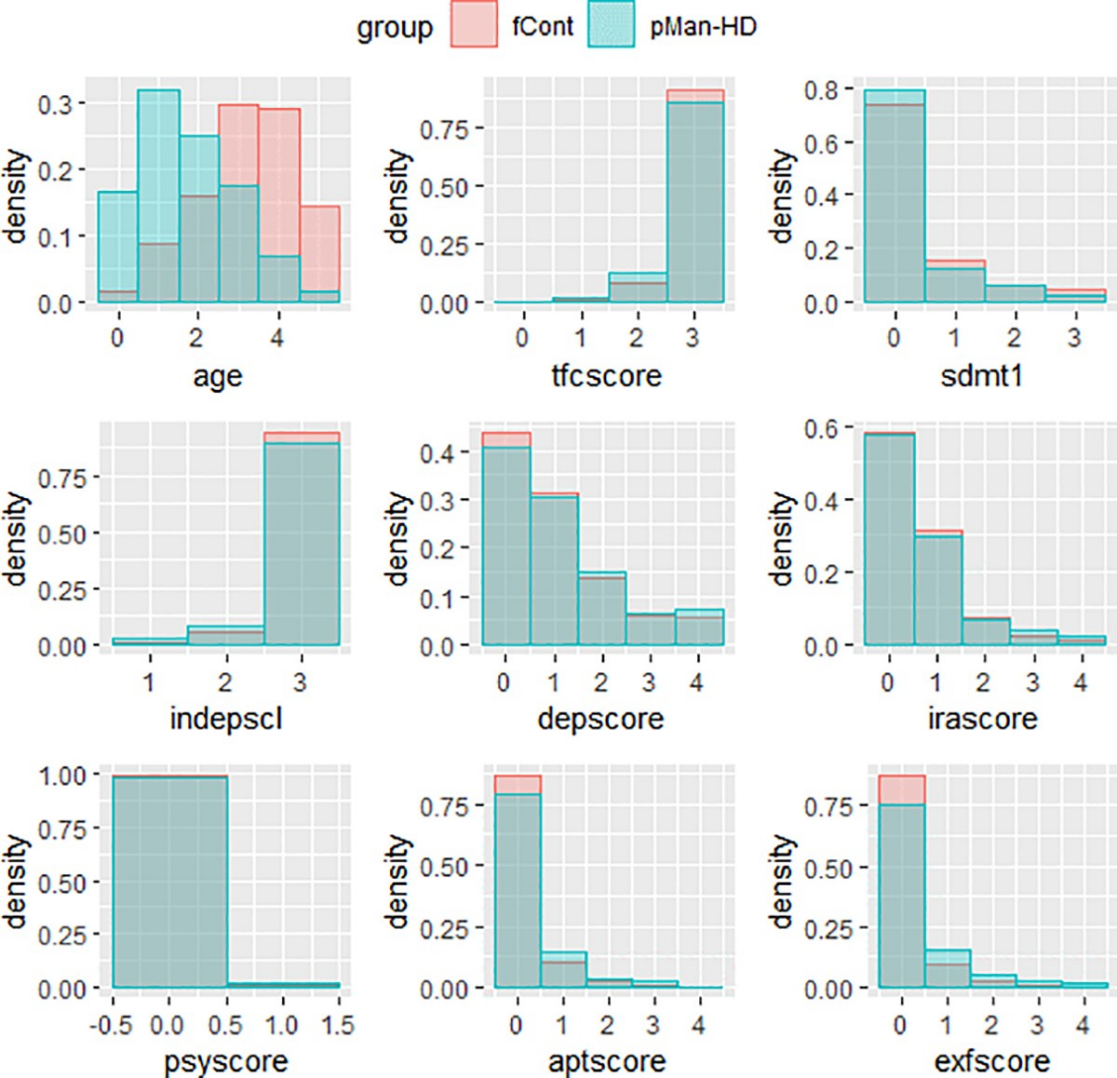
Normalised distribution (density) of the categorized factors (Table 1) within the pMan-HD and fCont subjects.

Table 1 summarizes the multi-scale categories for each factor as transformed using knowledge from clinical team of HD experts as well as the literature detailed in [29]. The weight-loss is categorized as -3 (severe weight-loss) to +3 (severe weight gain) as with the case of other factors as detailed in Table 1. The final data representations for the pMan-HD and fCont-HD contains the uniform representations of all factors (i.e., categorical dataset) forming the KB which is forwarded to pattern analysis and association mining. Fig 3 shows the normalised frequency distributions of the categorical factors within the pMan-HD and fCont-HD subjects. It can be noticed that different factors have varying number of categories while mostly representing the normal (i.e., healthy) distributions. Both groups (fCont-HD and pMan-HD) demonstrate some degrees of severity in domains such as depression, irritability, apathy and executive function, but in slightly different frequencies. These can be used to investigate their associations with weight-loss in HD.

**Table 1:** Clinical domain expert knowledge based data transformation (numeric to categorized form).

Factors	Expert knowledge-based categories of each factor					
**Age**	**Age<30**	**30≤ age <40**	**40≤ age <50**	**50≤ age <60**	**60≤ age <70**	**age ≥70**
	0	1	2	3	4	5
**wtCat**		**0≤ Δw <-5**	**-5≤ Δw <-10**	**-10≤ Δw <-20**	**-20 ≤ Δw**	
**(weight categories)**		0 (normal)	-1 (moderate)	-2 (severe)	-3 (very severe)	
**mmsetotal**	**25 ≤ mmse**		**20≤ mmse <25**	**10≤ mmse <20**	**mmse <10**	
	0		1	2	3	
**indepscl**	**0–40**		**41–80**	**81–95**	**≥95**	
	0 (severe)		1 (moderate)	2 (mild)	3 (normal)	
**fiscore**	**fiScore 0–5**	**fiScore 6–10**	**fiScore 11–15**	**fiScore 16–20**	**fiScore > 20**	
	0 (very severe)	1 (severe)	2 (moderate)	3 (mild)	4 (normal)	
**tfcscore**	**0–5**		**6–8**	**9–12**	**>12**	
	0 (severe)		1 (moderate)	2 (mild)	3 (normal)	
**motscore**	**Motscore = 0**	**Motscore: 1–31**	**Motscore: 32–62**	**Motscore: 63–93**	**Motscore > 93**	
	0 (normal)	1 (mild)	2 (moderate)	3 (severe)	4 (very severe)	
**exfscore**	**exfScore = 0**	**exfScore: 1–4**	**exfScore: 5–8**	**exfScore: 9–12**	**exfScore > 12**	
	0 (normal)	1 (mild)	2 (moderate)	3 (severe)	4 (very severe)	
**aptscore**	**aptScore = 0**	**aptScore: 1–4**	**aptScore: 5–8**	**aptScore: 9–12**	**aptScore > 12**	
	0 (normal)	1 (mild)	2 (moderate)	3 (severe)	4 (very severe)	
**irascore**	**iraScore = 0**	**iraScore: 1–4**	**iraScore: 5–8**	**iraScore: 9–12**	**iraScore > 12**	
	0 (normal)	1 (mild)	2 (moderate)	3 (severe)	4 (very severe)	
**depscore**	**depScore = 0**	**depScore: 1–4**	**depScore: 5–8**	**depScore: 9–12**	**depScore > 12**	
	0 (normal)	1 (mild)	2 (moderate)	3 (severe)	4 (very severe)	
**psyscore**	**psyScore = 0**	**psyScore: 1–4**	**psyScore: 5–8**	**psyScore: 9–12**	**psyScore > 12**	
	0 (normal)	1 (mild)	2 (moderate)	3 (severe)	4 (very severe)	
**sdmt1**	**sdmt1>42**		**sdmt1: 35–42**	**sdmt1: 27–34**	**sdmt1: 0–25**	
	0 (normal)		1 (mild)	2 (moderate)	3 (severe)	

### 2.3 Pattern analysis and rule mining

Analyzing the associations and patterns in high-dimensional datasets using conventional statistical approaches is impractical. The employment of machine intelligence for the analysis of complex dataset and identification of patterns, have been significantly increased in various domains such as medical imaging [30, 31], hand and facial gesture classification [32–34], eHealth systems [35], unstructured medical data analysis [36, 37] and many more. The pMan-HD in this study, contains 11 non-motor factors (see Table 1) each comprising multiple categories making it more complicated to be analysed by the human experts or conventional statistical approaches. The issue can be handled using intelligent pattern recognition algorithms such as Self-Organizing Maps (SOM) which are the unsupervised form of artificial neural networks forming a non-linear projection of a high-dimensional space on a lower-dimensions (typically 2-dimensioanl space) map [38]. SOM uses the competitive learning to preserve the topological properties of an input space which is different from error minimization algorithm used in other form of neural networks. The two-dimensional map representation is useful for pattern identification within the high dimensional data such as in this study. During the competitive learning phase, the input data samples (i.e., one subject’s record in our study) are iteratively mapped to SOM where a winning neuron (also called best matching unit) is identified based on the distance from its weights and the input vector (i.e., one sample/record from the dataset). The weight update is performed within the specific neighborhood radius resulting in similar samples mapped closely together. In summary, the SOM algorithm contains three major components that include: i) distance calculation between the input and weight vector with time complexity O(N^2^ * S), ii) identifying the winning neuron with complexity O(N * (S * log(S)), and iii) weight update using the neighborhood function with complexity of O(N^2^ * S). Where, N and S represent the number of samples (i.e., dataset records) and target classes (i.e., output neurons) respectively. Despite the categoric formation of the dataset in this study, its numeric property is preserved to be used for the competitive learning-based clustering algorithms. Detailed explanation and mathematical formulation of SOM can be found in [38].

**Fig 4:**
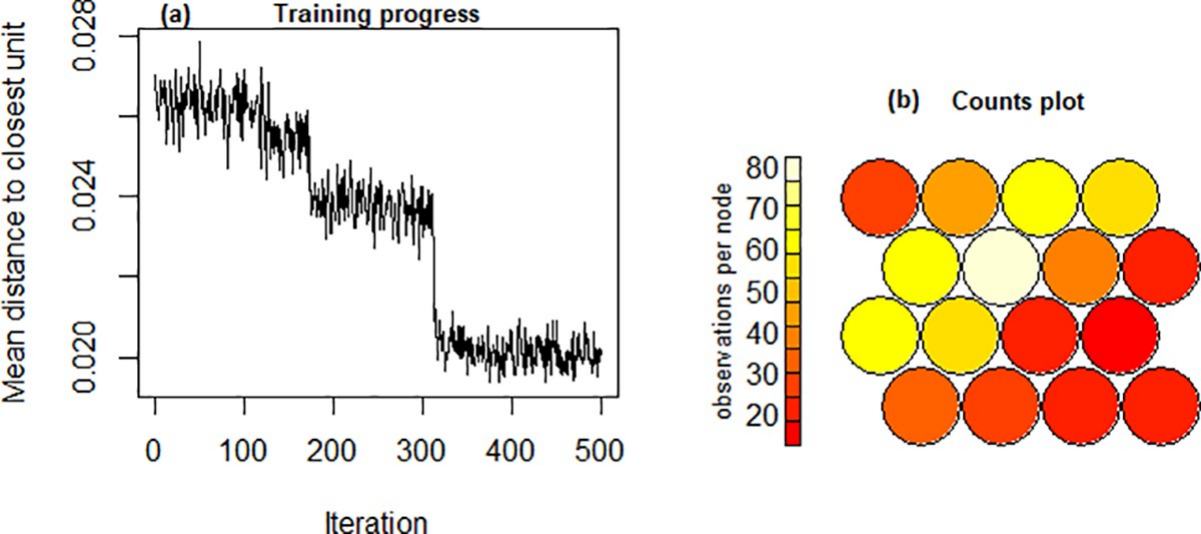
**(a).** SOM convergence during the training. **(b).** Count plot representing the records per node in SOM map for pMan-HD data.

Fig 4(A) shows the SOM training progression and convergence while Fig 4(B) show the count plot for pMan-HD data representing the sample distribution across the nodes within the SOM map. The count plot relates to the quality of SOM model indicating a varying distribution of samples (20–80 samples in this case) over the map without empty nodes. The distribution of the dataset (samples and variables) across the SOM map can be visualised in two-dimensional space using the heatmap which is one of the most useful property of SOM. In this work, the heatmap is employed to identify the inter-relationships between multiple factors within the pMan-HD and fCont-HD data.

**Fig 5:**
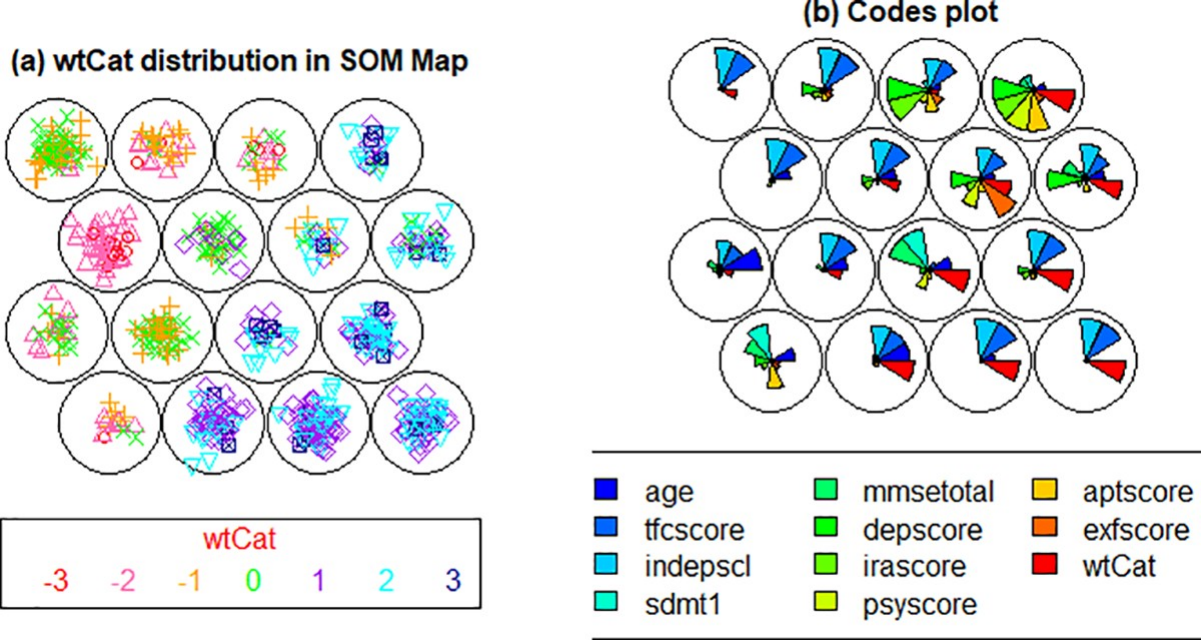
**(a).** Distribution of wtCat (-3 to +3) across the SOM nodes in pMan-HD subjects, **(b).** Codes plot distribution of combined factors in SOM nodes. The node positions start from bottom left (node 1) and ends at top-right (node 16).

Fig 5(A) demonstrates the wtCat distribution across the SOM nodes in pMan-HD subjects where each sample of dataset in a node, represents a subjects’ clinical record for the selected factors. The algorithm automatically organizes the map (i.e., position of samples and nodes) based on Euclidean distance between the codebook vectors of neurons/nodes. In other words, similar samples get together within same node. Likewise, nodes with high similarity (i.e., nodes with less neighboring distance) get together within the map whereas, dissimilar nodes get far from each other. As an example, most of the subjects with severe weight-loss (i.e., wtCat -3, -2) are pushed towards left positions (i.e., node 1, 4, 9, 14, 15) in Fig 5(A) representing the similar behavior of wtCat factor in these subjects. On the other hand, most of the high weight gaining (wtCat +3, +2) samples pushed themselves towards the right-side nodes (e.g., nodes 2, 3, 4, 7, 8, 11, 12, 16) in the map. This distribution clearly indicates the distinctive behavior of weight-loss in pMan-HD subjects that needs further investigation in terms of its associations with other biological factors. Fig 5(B) shows the codes plot which indicates a very useful information about the combined behaviour of multiple factors across the SOM map. It can be observed that the combined distribution is diverse across the map specifically in terms of wtCat -3, -2. For instance, node 9 representing the severe weight-loss comprises medium age, high tfcscore and indepscl whereas, node 1 indicate higher categories for sdmt and aptscore while comparatively younger age group. Likewise, node 15 (representing the weight-loss) comprises high depscore and irascore in combination with younger age subjects.

**Fig 6:**
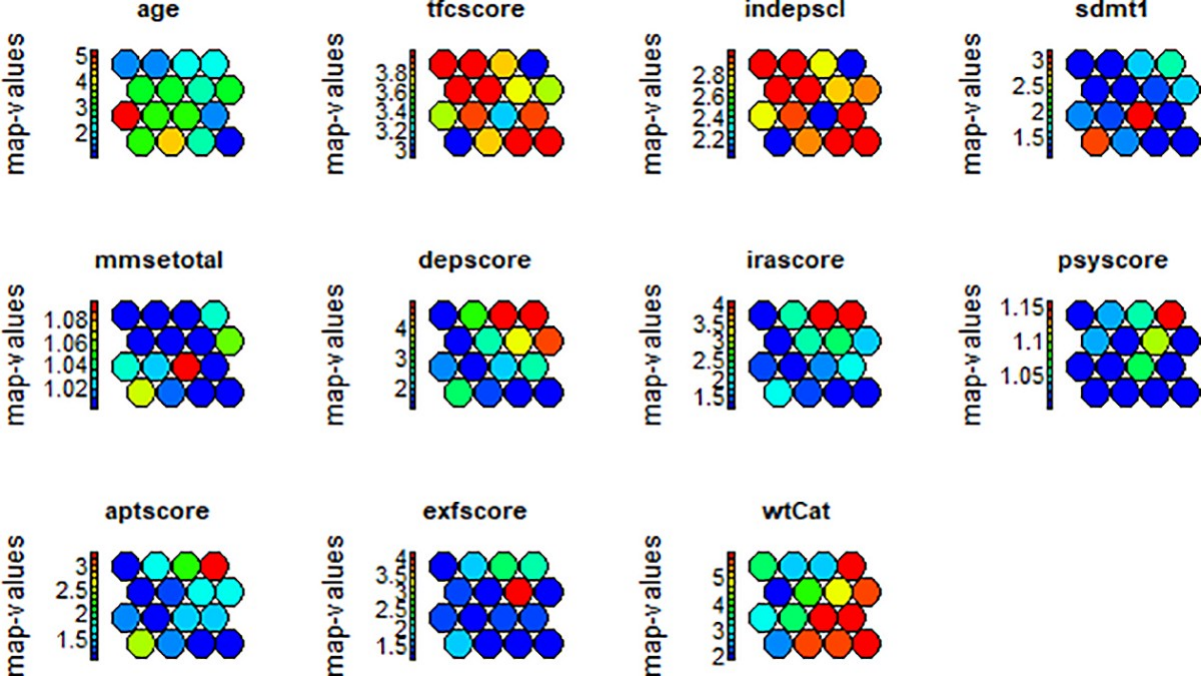
Heat maps representing the distribution of individual factors in pMan-HD across the SOM map.

Fig 6 further shows the distribution of individual factors across the SOM heat maps for pMan-HD subjects. Firstly, the map indicates the overlapping distributions for tfcscore with indepscl and depscore with irascore. This indicates correlation between these factors within the pMan-HD subjects. Secondly, the heat maps indicate the relationship between each individual variable and wtCat in pMan-HD. As an example, tfcscore and indepscl indicate high categories for the severe weight-loss (i.e., wtCat -3, -2) whereas psyscore and exfscore indicate an opposite behavior. On the other hand, age, depscore, irascore and sdmt indicate obscure behavior which is needed to be further investigated.

**Fig 7:**
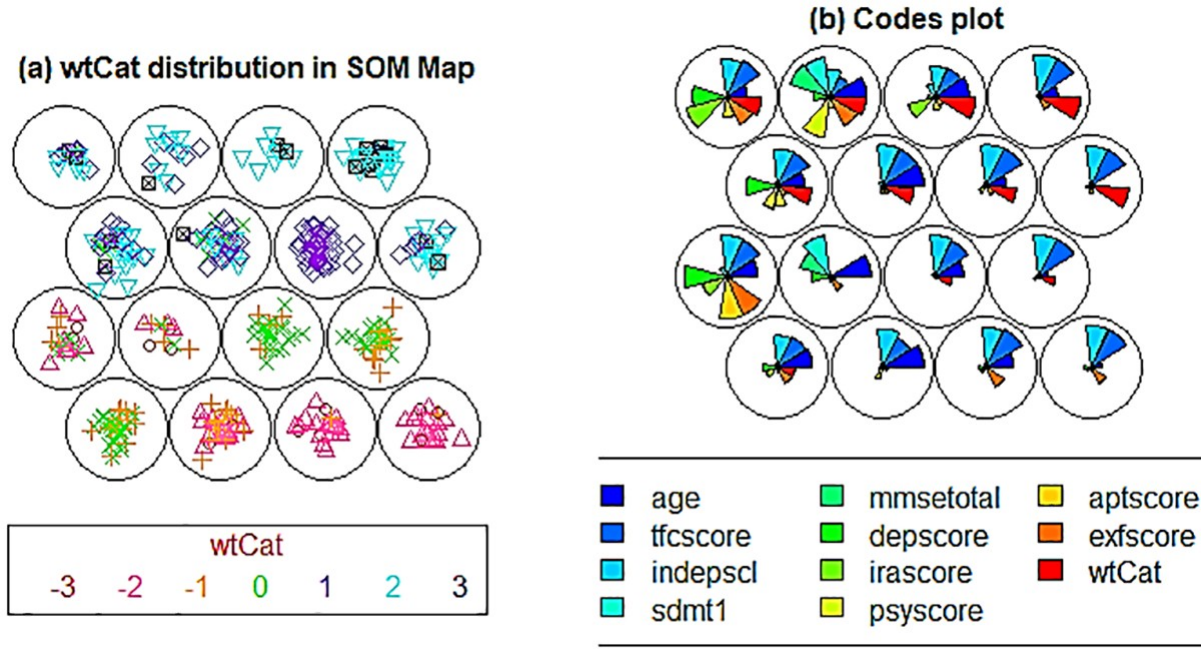
**(a).** Distribution of wtCat (-3 to +3) across the SOM nodes in fCont-HD subjects, **(b).** Codes plot indicating the distribution of combined factors (fCont-HD subjects) in SOM nodes.

The distribution of wtCat and combined factors in fCont-HD dataset is shown in Fig 7(A) and 7(B) respectively. It can be seen that the distributions of fCont-HD factors are different when compared with the pMan-HD (Fig 6) indicating the varying inter-class associations in both groups. Specifically, node 2, 3 (comprising wtCat -3, -2) indicate the presence of exfscore with tfcscore and indepscl. Likewise, node 5, 6 indicate combinations of different factors as compared to pMan-HD map. In summary, the SOM based lower-dimensional visualization clearly indicate the variations in factors’ behavior and distributions in both groups (i.e., pMan-HD and fCont-HD) that needs further investigation to explore the discrete inter-relationships specifically between the psychiatric factors (such as irritability, sdmt, depression, apathy) and wtCat (-3, -2).

For this purpose, we utilize the Class Association Rules (CARs) which is the special case of conventional rule mining [39] where the target class is only used as a consequent of rule. The CARs have been utilized in various domains including healthcare, dimensionality reduction, education and decision making [40–44]. One of the major uses of CARs is the identification of frequent patterns within the large dataset that can easily be interpreted by the human experts in form of rules. Let *‘F’be* the list of factors defined in Table 1 containing *T = {t*_*1*_, *t*_*2*_, *t*_*3*_, *… t*_*N*_*}* observations (i.e. subjects’ clinical records) in the dataset where each observation ‘*t*_*i*_’ containing a subset of factors *‘F’*. The X→Y relationship in CARs indicates the disjoint itemset i.e., X∩Y = ∅ occurring in the *T* (i.e., dataset) as *antecedents* and *consequents* respectively. An important property of a rule is the corresponding *support count* (*σ*) representing the number of observations containing that itemset (factor/s) and can be formulated as:

$$\left(X)=|\left\{t_{i}|X\subseteq t_{i},t_{i}\in T\right\}\right|$$ (2)

The strength of a rule (X) and hence the association are usually controlled by using *confidence (c)* and *support (s)* metrics where:

$$s(X\Rightarrow Y)=\frac{\sigma (X⋃Y)}{N}$$ (3)

where *N* represents the total number of observations/records within the dataset. The confidence of a rule *c* represents the percentage for which factor *Y* occurs with the presence of factor *X* and is represented as:

$$c(X\Rightarrow Y)=\frac{\sigma (X⋃Y)}{\sigma (X)}$$ (4)

The strength of a rule usually identified by varying thresholds for ‘*s*’ and ‘*c*’ [45]. However, the significance/importance of an association may be misinterpreted by these metrics. This is because it only accounts for how popular the X is but not the Y. If the X occurs very frequently, there will be a higher chance that an observation in the dataset containing the X will also contain the Y, thus inflating the value of ‘*c*’ [45]. To account for the base popularity of both constituent items (i.e., X and Y), a third measure called *lift* is used that measures the correlation between X and Y of a rule, indicating the effect of the X on the Y, and is calculated as:

$$lift(X\Rightarrow Y)=\frac{s(X\cup Y)}{s(X)*s(Y)}$$ (5)

A value of *lift*(X⇒Y) = 1 indicates independence between *antecedents* and *consequent*, whereas *lift*(X⇒Y)>1 indicates positive dependence of X and Y. We further deploy additional measures to validate the strength of extracted rules. For instance, *Conviction* is a probabilistic measure that incorporate the rules direction (an alternative to *lift*) with range 1 to ∞ (1 indicate independence between X and Y). Chi-square metric and associated *p-value* represents the confidence level of dependency between antecedents and consequent of a rule. Likewise, we deployed the *Strength* measure generating 0 for negative correlation, ∞ for perfectly positive correlations and 1 for independent X and Y. Detailed explanation about these metrics can be found in [46].

To investigate the associations between specific factors (e.g., *iraScore*, *sdmt*, *depScore* etc.) as *antecedents* and the *consequent* (*wtCat -3* and *wtCat -2*) in this study, we utilise the CARs and Apriori algorithm (with the computational complexity of O(2^N^) where N is the width of our dataset) which are the well-established methods to identify the frequent patterns within the dataset in the form of human understandable rules. A detailed explanation about CARs and the Apriori algorithm can be found in [31]. The major challenge with the conventional rule mining is the generation of high number of rules that is impractical to be interpreted by conventional approaches or human expert. However, this issue is resolved using sequential filtration of irrelevant rules with varying threshold values for ‘*c*’ and ‘*s*’. Selection of optimum value for these thresholds entirely depends upon the nature of problem and data itself [47]. Based on imperical experiments, we perform the rule filtration while optimising several parameters that include confidence (minimum confidence = 0.9), minimum length = 2, maximum length = 5 resulting the extraction of only highly associated and compact list of rules. As per research question in this study, we extract conditional rules based on wtCat (-3 and -2) as consequent that further limits the generation of large set of rules. In addition, we utilise the redundant rules elimination [48] to filter the unecessary noise and therefore, resulting the list of representative rules that can easily be interpreted by the human experts.

To further validate the SOM and CARs based outcomes, we deploy the *Mutual Information* (*I*) which uses the *Conditional Entropy C*(*H*) indicating the uncertainty of a variable when other is known and is given by:

$$C(H)=\sum_{i}p(X=x_{i})H(Y|X=x_{i})$$ (6)

Where X and Y are random variables, *p* is the probabilty of known variable (X in this case) and *H* is the entropy of Y conditioned on X. The *I* can be expressed as:

I(X;Y)=H(Y)−H(Y|X) (7)

Where *I*(Y; X) measures the reduction of uncertainty on on Y (or X) due to the other variable X (or Y). Both, *C(H)* and *I* can be used to test the conditional independence between multiple variables. For instance, higher *I* indicates higher dependency between two variables. Further details and mathematical formulation of *C(H)* and *I* can be found in [49].

## 3. Experimental results

In order to investigate the potential class associations between the non-motor factors and weight-loss in HD in premanifest stage, experiments are conducted using the KB categorised pMan-HD and fCont-HD datasets comprising selected factors described in Table 1. The CARs algorithm is used with the parametric configurations and rule filtration explained in Section 2.3 while considering the listed factors as antecedents and target wtCat as consequent for both pMan-HD and fCont-HD. The specific focus of the experiments is to analyze the significant associations between the severe weight-loss (i.e., subjects with wtCat -3, -2) and certain factors particularly the psychiatric factors (such as irritability, depression, sdmt) and functional abilities in both groups.

**Fig 8:**
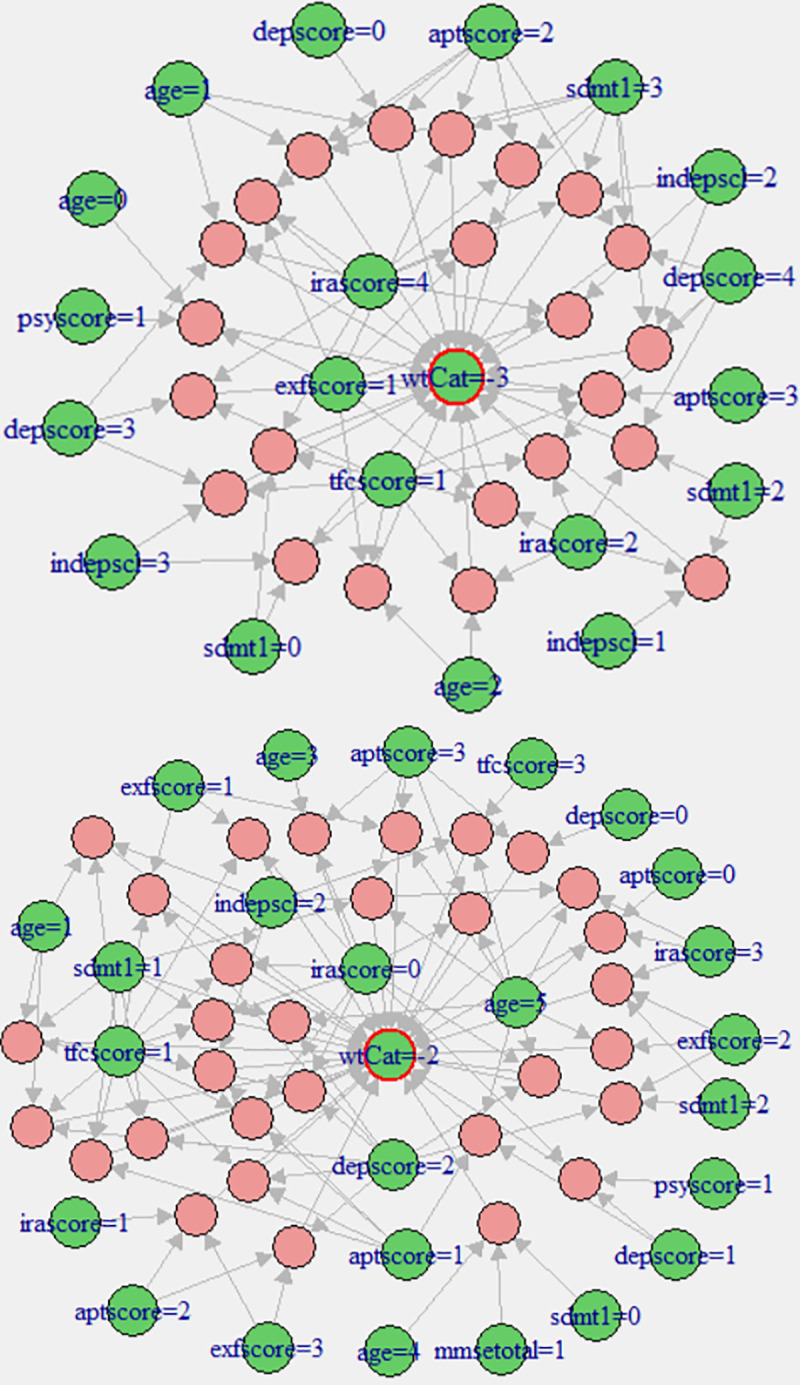
**(a).** Visualisation of representative associations between multiple factors and wtCat in preMan-HD (wtCat = -3)**. (b).** Visualisation of representative associations between multiple factors and wtCat in preMan-HD (wtCat = -2).

Fig 8(A) demonstrates the 31 representative rules (shown as red circles) indicating the list of factors highly associated with the severe weight lost (wtCat = -3) in the pMan-HD. The size and colour intensity (i.e., red colour) of the circles relate to the relative strength of the rule in terms of confidence and lift measure respectively. These non-redundant rules indicated high association between wtCat = -3 and multiple factors such as moderate tfscore (1), mildly affected exfscore (1), moderate aptscore (2), severe sdmt (3) and severe irascore (4). In a similar way, Fig 8(B) shows the associations for wtCat = -2 within the pMan-HD data. The plot clearly indicates the excessive occurrence of high age (i.e., age = 5) subjects despite normal to mild aptscore, sdmt and irascore. It also demonstrates an association of wtCat -2 with high aptscore and exescore (2,3) or moderate depscores (1,2). The plot also demonstrates that wtCat-2 is associated with mild-moderate scores on sdmt (0,1).

Table 2 presents antecedents’ list for the rules shown in Fig 8(A). The statistical metrics validate the strength of these associations indicating confidence >0.9, lift ≥125, conviction = ∞, Chi-squared >124, p-value <7.4 e-29, and strength >1125. The p-value<<0.05 clearly indicates the significant dependence between the antecedents and consequent (wtCat = -3) listed in Table 2. As mentioned earlier (in Section 2.3), elimination of redundant rules and constraints over length of antecedents reduces the number of rules to the best representative rules making them more easily understandable and interpretable by humans. Outcomes in Table 2 indicate the high representation of moderate to severe iraScore (2, 4), depScore (3, 4) and tfscore (1) as well as severe scores on sdmt (2,3) when the antecedent is set to wtCat -3 within the pMan-HD. It can also be noted that the list contains a relatively young age group (0–2) associated to wtCat -3. Furthermore, it can be seen that the association of wtCat -3 with moderate-severe sdmt appears in the same nonredundant rule as high depscore and irascores or in rules when there is high aptscores. On the other hand, exfscore remains normal to mildly affected in most of the cases.

**Table 2:** Antecedents in rules with high association between selected factors and severe weight-loss with wtCat = -3 (consequent) in pMan-HD.

tfcscore = 1,depscore = 3,irascore = 4	sdmt1 = 3,irascore = 4,exfscore = 1
tfcscore = 1,sdmt1 = 0,irascore = 4	irascore = 4,aptscore = 2,exfscore = 1
tfcscore = 1,aptscore = 3,exfscore = 1	age = 1,depscore = 3,irascore = 4
tfcscore = 1,indepscl = 3,depscore = 3	indepscl = 2,irascore = 4,exfscore = 1
tfcscore = 1,depscore = 4,irascore = 2	sdmt1 = 3,depscore = 4,aptscore = 2
tfcscore = 1,irascore = 2,exfscore = 1	sdmt1 = 3,aptscore = 2,exfscore = 1
age = 2,tfcscore = 1,irascore = 2	age = 1,sdmt1 = 3,aptscore = 2
age = 2,tfcscore = 1,exfscore = 1	indepscl = 2,sdmt1 = 3,depscore = 4
tfcscore = 1,indepscl = 3,sdmt1 = 0	indepscl = 1,sdmt1 = 2,irascore = 2
age = 0,psyscore = 1,exfscore = 1	age = 1,depscore = 0,aptscore = 2
sdmt1 = 3,irascore = 4,aptscore = 2	sdmt1 = 2,depscore = 4,irascore = 2
indepscl = 2,sdmt1 = 3,irascore = 4	

Table 3 presents the descriptive form of representative rules (antecedents only) shown in Fig 8(B) with confidence >0.9, lift ≥27, conviction = ∞, Chi-squared >26, p-value <3.2e-07, and strength >990. Similar to wtCat(-3) in pMan-HD subjects, those rules indicated strong association between wtCat -2 and severe tfcscore (1) that occurs in combination with one of the mild-moderate factors depscore, aptscore or sdmt in this case. On the other hand, irascore indicates normal to mild measures across the rules except the cases where aptscore is normal and age group is high(5) or in combination of moderated sdmt and exfscore. One of the interesting aspects in Table 3 is the inverse behaviour indicated by aptscore and irascore (within high age group) when appear together as antecedents. A high irascore appears in combination with the normal-mild aptscore and vice versa. It is also worth noting that, sdmt, irascore and exfscore indicate similar behaviour when combined together as antecedents. In summary, the outcomes in Tables 2 and 3 indicate that with the severity levels of irascore, sdmt and depscore increase with the progression of weight-loss from wtCat -2 to wtCat -3.

**Table 3:** Antecedents in rules with high association between selected factors and severe weight-loss with wtCat = -2 (consequent) in pMan-HD.

age = 5,exfscore = 2	tfcscore = 1,irascore = 0,exfscore = 1
depscore = 0,aptscore = 3	age = 5,sdmt1 = 1,aptscore = 3
age = 4,sdmt1 = 0,mmsetotal = 1	age = 5,aptscore = 3,exfscore = 1
tfcscore = 1,indepscl = 2,aptscore = 1	age = 5,irascore = 0,aptscore = 3
age = 1,tfcscore = 1,indepscl = 2	age = 5,indepscl = 2,irascore = 3
tfcscore = 1,indepscl = 2,irascore = 0	age = 5,irascore = 3,aptscore = 0
tfcscore = 1,sdmt1 = 1,aptscore = 1	age = 5,tfcscore = 3,indepscl = 2
tfcscore = 1,sdmt1 = 1,depscore = 2	age = 5,sdmt1 = 1,depscore = 2
age = 1,tfcscore = 1,sdmt1 = 1	age = 5,depscore = 1,aptscore = 1
tfcscore = 1,sdmt1 = 1,irascore = 0	age = 5,depscore = 2,irascore = 0
tfcscore = 1,depscore = 2,aptscore = 1	depscore = 1,irascore = 0,psyscore = 1
tfcscore = 1,irascore = 0,aptscore = 1	depscore = 2,aptscore = 2,exfscore = 3
tfcscore = 1,depscore = 2,exfscore = 1	irascore = 1,aptscore = 2,exfscore = 3
age = 1,tfcscore = 1,depscore = 2	age = 3,irascore = 0,aptscore = 3
tfcscore = 1,depscore = 2,irascore = 0	sdmt1 = 2,irascore = 3,exfscore = 2
{sdmt1 = 2,depscore = 2,exfscore = 2}	

**Fig 9:**
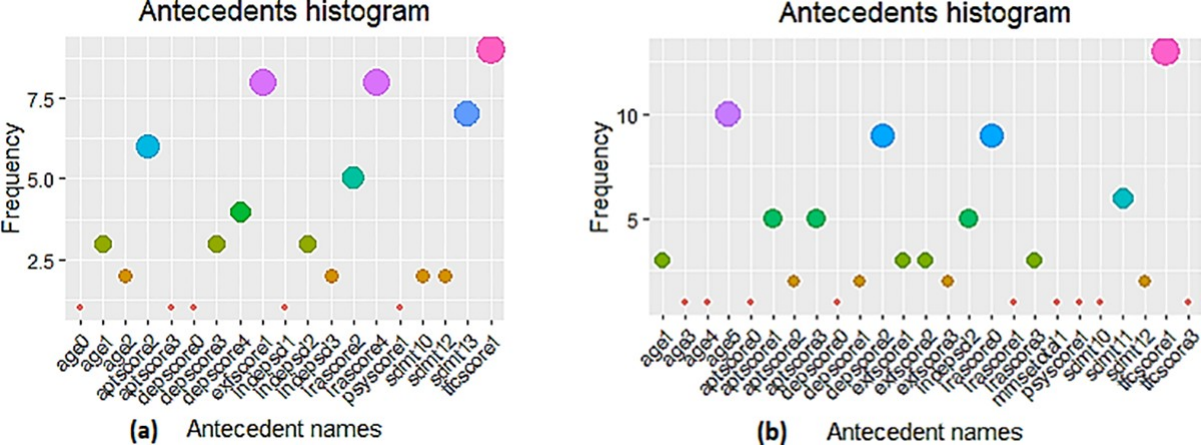
**(a).** Antecedents’ histogram in CARs for pMan-HD subjects indicating individual factors’ frequency for wtCat -3. **(b).** Antecedents’ histogram in CARs for pMan-HD subjects indicating individual factors’ frequency for wtCat -2.

Although we utilised the rule filtration with parametric and non-redundancy constraints, it is useful to present the antecedents’ information (i.e., rules) in a simpler form to better examine the features individually. For this purpose, we extracted the frequency histograms within the antecedents of rules as shown in Fig 9. The frequency histograms help to visualise more complex and large set of rules to further investigate the significance of individual factors within the list of representative rules. However, it is important to consider the associations when antecedents are combined with other factors (i.e., how the association varies with varying combinations in antecedents). Fig 9 presents the antecedents’ histogram for the representative rules within the pMan-HD reflecting the Fig 8(A) and 8(B). These outcomes are aligned with the aforementioned rules (Tables 2 and 3) indicating a mildly effected exfscore and moderate to severe irascore, tfcscore, sdmt and depscore in wtCat -3. Likewise, tfcscore and exfscore in wtCat -2 indicated slight change in behaviour in contrast to a decrease in the severity level of depscore, sdmt and irascore which indicates the combined progression in weight-loss severity and these factors.

Furthermore, the CARs outcomes are also aligned with SOM based distributions shown in Figs 5 and 6. For instance, node 9, 14, 15 presents the low age distribution for wtCat -3 as compared to node 1, 5 with moderate to severe age group for wtCat -2 that aligns with the CARs outcomes. Likewise, high irascore and depscore appear in node 15 while normal to normal to mild sdmt, aptscore appeared in most of the nodes with wtCat -2 which also aligns with the CARs outcomes. However, as the distribution of wtCat comprises mixed occurrences within the SOM map, the CARs produce comparatively discrete level information while conditioning the target factor and constraints on antecedents.

Various similar works presented in Table 4 addressed the diverse aspects of the HD factors at different stages sing different conventional statistical tools. For instance [15], reported that the weight-loss in HD patients occurs with the disease progression which is not the case otherwise (i.e., control group) [55]. reported the high prevalence of neuropsychiatric symptoms such as apathy, irritability, and executive dysfunction in HD specifically at premanifest stage. However, despite the evidence of weight-loss over the disease progression, none of the existing studies addressed the weight-loss relationship with the psychiatric features as in the present work. Likewise, these works utilised the conventional statistical methods that are impractical to identify the multi-dimensional associations within the complex data as well as effective visualisation of the useful patterns within the complex data. In contrast, we employed the SOM and CARs to help visualising and interpreting the complex patterns that are not possible with the tools used by existing works specifically, producing human understandable rules and low-dimensional visualisations of complex patterns.

**Table 4:** Comparison of proposed study and existing works in this domain in terms of target variable, statistical algorithms, level of significance and explaining attributes.

Study	Factors	Dependent variable	Level of significance	Method	Disease stage
[11]	Annualized disease progression	quantitative imaging cognitive scores motor scores	High	Linear regression	Premanifest and early manifest HD
[15]	Dietary, food quantity	Weight gain/loss	High	Student t-test	manifest
[23]	CAG repeats	Weight loss	High	Mixed effects model analysis	manifest
[25]	Weight loss, Non-motor features (sleep, apathy)	Progression over time	N/A	Review of literature	Manifest
[53]	Psychiatric symptoms	Progression over time	High	Linear mixed effect regression models	Premanifest and manifest
[54]	Depression, Irritability, Apathy	Progression over time	Mild-High	Multivariate linear regression analysis	Mutation carriers/premanifest
[55]	Neuropsychiatric scale (PBA)	Comparison of Neuropsychiatric symptoms between different groups	Moderate-High	Odds ratio calculation-logistic regression analysis	Premanifest
Our work	Cognitive scales, functional score, psychiatric assessments	Weight loss	Moderate-High	Self-Organising Maps, Class Rule Mining, Chi-square test, mutual information	Premanifest

We further deploy the commonly used Chi-square test of independence and mutual information *I* (Eqs 6 and 7) to investigate the conditional dependence between multiple factors and severe weight loss (wtCat -3, -2) in pMan-HD. This analysis might be useful to validate the outcomes retrieved through CARs based generated rules in Table 3. Table 5 presents the ‘*I*’ measure and test of independence outcomes between wtCat and other factors within the pMan-HD data. It can be noticed that *I* measure is comparatively higher for age, tfscore, depscore, irascore and aptscore for pMan-HD subjects. Likewise, the higher Chi-square value ($\bar{\chi }$) and corresponding p-values<0.05 (i.e., 95% confidence level) are also aligned with *I* measure for these factors and indicate the significance of inter-dependence between these factors and weight loss. These outcomes also align with most of the CARs rules specifically the histograms presented in Fig 9. However, it is important to note that the statistical measure in Table 5 does not consider the attribute interactions and multiple combinations. For instance, the combined *I* for some factors (e.g., age, depscore, irascore) is significantly larger (*I* = 0.21) than corresponding individual *I* measures in Table 5 (*I* = 0.04) which clearly indicate the impact of multiple combinations within the wtCat-HD data. On the other hand, the CARs based representative rules in Tables 2 and 3 are able to demonstrate the multiple combined factors (identified as significantly associated with severe weight loss) based on various statistical measure including $\bar{\chi }$, *p-value*, *lift* and *conviction* metrics.

**Table 5:** Conditional information gain and Chi-square test of independence outcomes for weight-loss and other factors in pMan-HD and fCont-HD subject.

Factor (F)	pMan-HD Subjects			fCont-HD Subjects		
	I(wtCat)|F	$\bar{\chi }$	p-value	I(wtCat)|F	$\bar{\chi }$	p-value
age	**0.024**	36.7	0.18	0.020	**65.2**	**0.00**
tfcscore	**0.022**	**32.7**	**0.01**	**0.049**	**33.1**	**0.01**
indepscl	0.008	22.8	**0.02**	**0.029**	17.4	0.13
sdmt1	0.025	21.5	0.25	**0.042**	19.4	0.36
mmsetotal	0.002	3.2	0.77	0.026	**24.7**	**0.01**
depscore	**0.066**	**40.2**	**0.02**	**0.032**	16.9	0.85
irascore	**0.045**	**41.2**	**0.01**	0.029	26.4	0.33
psyscore	0.006	9.4	0.14	0.001	6.5	0.36
aptscore	**0.027**	**35**	**0.06**	0.003	20.3	0.67
exfscore	0.020	28	0.25	**0.029**	**45.4**	**0.00**

**Fig 10:**
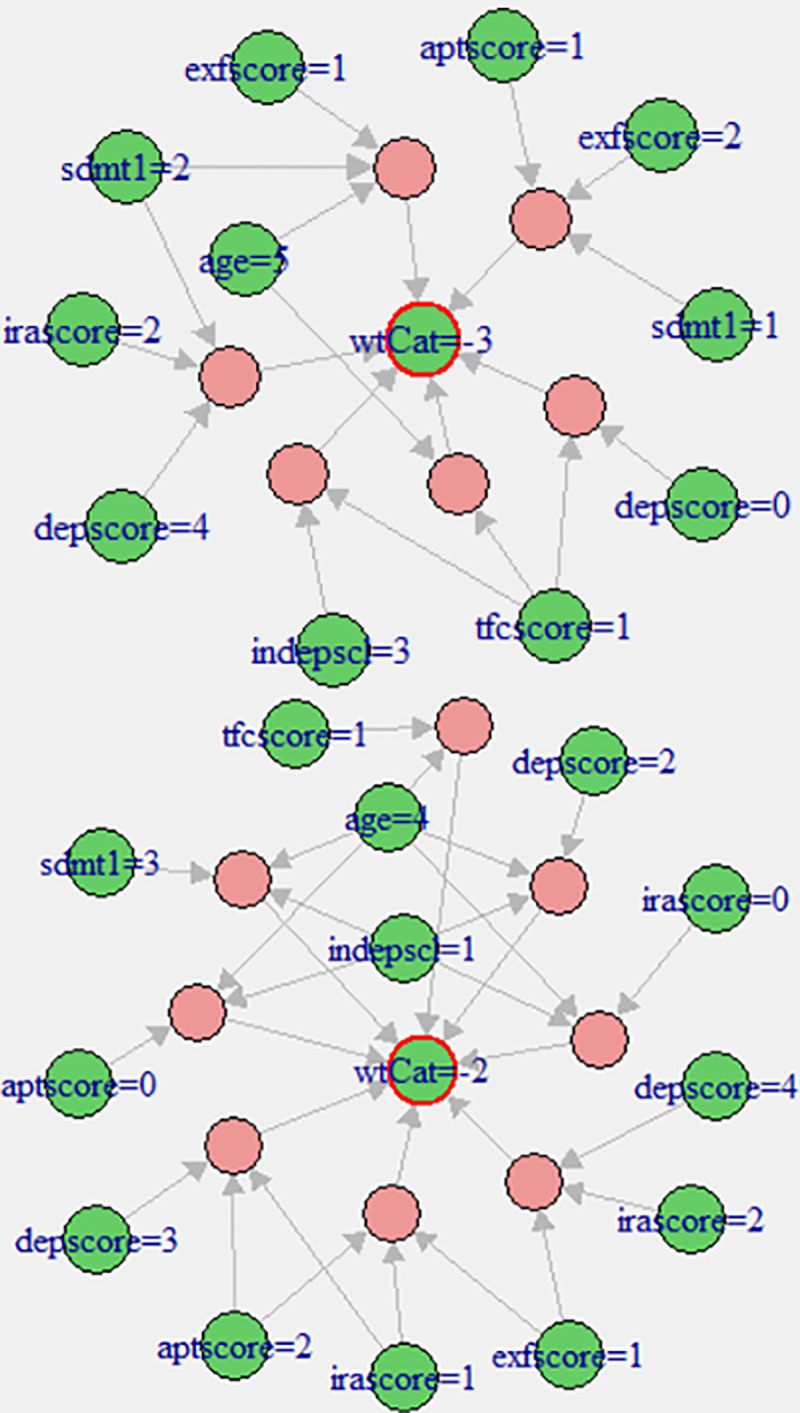
**(a).** Visualisation of representative associations between multiple factors and wtCat in fCont-HD for wtCat = -3. **(b).** Visualisation of representative associations between multiple factors and wtCat in fCont-HD for wtCat -2.

Fig 10(A) summarises the representative rules for high associations between the wtCat -3 and rest of the factors within the fCont-HD data. In contrast to pMan-HD, the irascore and sdmt being mild to moderate indicated high associations with wtCat -3 while depscore is varying from normal to severe levels. On the other hand, tfscore shows similar behaviour to pMan-HD indicating the high association between severe weight-loss and severe tsfscore. Furthermore, the results indicate high degree of association between older ages and sever weight-loss in fCont-HD.

Fig 10(B) show the representative associations (confidence,1 and lift ≥ 21) between wtCat -2 and other factors in fCont-HD. The outcomes indicate that a high depscore appears with moderate irascore or aptscore in wtCat -2. Whereas high age group (4) and moderate indepscore indicate strong association with wtCat -2 in fCont subjects. Table 6 presents the antecedents in representative rules shown in Fig 10(A) and 10(B) in descriptive form.

**Table 6:** Antecedents in rules with high association between selected factors and severe weight-loss (wtCat -3, -2) in fCont-HD.

Consequent (wtCat) = -3	Consequent (wtCat) = -2
age = 5,tfcscore = 1	age = 4,tfcscore = 1
tfcscore = 1,depscore = 0	age = 4,indepscl = 1,sdmt1 = 3
tfcscore = 1,indepscl = 3	age = 4,indepscl = 1,depscore = 2
sdmt1 = 1,aptscore = 1,exfscore = 2	age = 4,indepscl = 1,irascore = 0
	age = 4,indepscl = 1,aptscore = 0
sdmt1 = 2,depscore = 4,irascore = 2	depscore = 3,irascore = 1,aptscore = 2
	irascore = 1,aptscore = 2,exfscore = 1
age = 5,sdmt1 = 2,exfscore = 1	depscore = 4,irascore = 2,exfscore = 1

**Fig 11:**
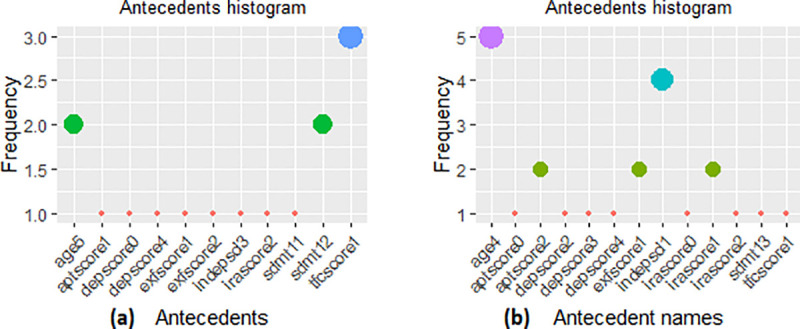
**(a).** Antecedents’ histogram in CARs for fCont-HD subjects indicating individual factors’ frequency for wtCat -3. **(b).** Antecedents’ histogram in CARs for fCont-HD subjects indicating individual factors’ frequency for wtCat -2.

Fig 11 presents the histogram of individual antecedents in representative associations within the fCont-HD subjects reflecting Fig 10(A) and 10(B). It can be observed that most of the high frequency antecedents here indicated normal to mild category except *depscore*, *tfcscore and indepscore*. These outcomes also overlapped significantly with the SOM based codes plot in Fig 7, which also indicate low-to-mild values for exfscore, sdmt1 and aptscore in most of the severe weight loss nodes (i.e. node 2 to 6).

Likewise, higher age group subjects appeared in most of the nodes representing wtCat -3, -2 while indepscl and tfcscore indicate varying distributions that also partially aligns with rules in Table 6. In addition, the Chi-square test of independence and conditional information gain values (shown in Table 5) further validate the outcomes of CARs (as shown in Table 6). For instance, $\bar{\chi }$, p-value and *I* measures indicate the significance of dependence between wtCat -3, -2 and several factors including depscore, exfscore, age, indepscl and tfcscore that align with the CARs outcomes. However, as previously mentioned, the major limitation of these methods (Chi-square test and Information gain) is lack of simultaneous factor analysis and discrete level information extraction for each factor. As an example, the joint mutual information *I(wtCat | tfcscore*, *sdmt1*, *depscore)* and *I(wtCat | age*, *sdmt1*, *depscore)* increases to 0.22 and 0.31 respectively as compared to 0.04 (maximum) for individual factors. This indicate that various combination of these factors might produce higher dependence with the severe weight-loss that will be useful to be further investigated from a clinical perspective. However, identification of optimal combination as well as factor level grained information extraction may be impractical with such methods. This overall show the leverage of CARs over conventional statistical measures to analyse the complex associations and dependencies between combined multiple factors at discrete level that can be understandable by humans.

## 4. Discussion

It has been established based on large scale studies such as PREDICT-HD and TRACK-HD [10–11] that HD’s non-motor factors (such as depression, irritability, and cognitive dysfunction) start years before the onset of motor symptoms that defines the diagnosis of HD. Depression is amongst the most common psychiatric factors in HD, even decades before the onset of the disease with the lifetime prevalence of major depression in 50% and above, compared to 15% in the normal population [50, 51]. Irritability is known to occur across all stages of the disease and studies have shown the prevalence to be 38–73% [52–54]. It is known to correlate with depression and increases in severity in premanifest subjects as they come closer to developing the motor stage [54]. Apathy can start early in the disease but is known to progress as the disease progresses. This is not the case with depression and irritability [10, 55]. Studies have shown that although CAG repeat correlates with age of onset and speed of deterioration, it does not necessarily correlate with the onset of severity of the psychiatric manifestations [51].

Whilst the unequivocal motor factors of HD are associated with pathology in the basal ganglia and cerebral cortex and cognitive dysfunction is associated with pathology in the cortex and the striatum parts of the brain, the underlying neurobiological mechanisms for these non-motor factors are not known [25]. Understanding these may lead to important insight into early disease mechanisms and hence aid in targeting therapy early on in the neurodegenerative process.

Studies have suggested the involvement of the hypothalamus early in HD and given its role in certain functions such as metabolism (weight control), and its involvement in early HD symptoms, but its role is still unclear [25]. In this sense, the proposed study is aimed at investigating the relationship of weight-loss and other non-motor factors in HD, in an attempt to shed light on their neurobiology. In contrast to existing works that use conventional statistical approaches (see Table 4), the proposed study might be useful for better visualisation of multi-dimensional dataset and analysis of complex patterns producing human understandable rules while utilising the clinical knowledge and machine intelligence.

The authors considered two categories of weight-loss: severe 10–20% of body weight (wtCat-2) and very severe >20% of body weight (wtCat-3) at 3–5 year follow up in premanifest HD subjects and family controls and studied its associations with cognitive, psychiatric and functional independence measures. The results demonstrated a strong association of the wtCat-3 with younger age group 1, 2 (30–50 years). This is in keeping with the established fact that younger onset is associated with severe form of the disease. It also showed a clear association of this category with moderate impairment on the total functional score when irritability and depression severity are high or when functional scores are moderately impaired tfcscore = 1, combined with moderate/high apathy scores and mild executive dysfunction scores. Interestingly from these rules of associations, it seems that subjects who are losing weight severely and are functionally moderately impaired have one of two predominant pictures: irritability and depression or apathy and executive dysfunction but not both. This is in keeping with studies highlighted previously [10, 50–55] that have shown an association of depression and irritability. Apathy on the other hand seems independent and possibly unrelated to irritability and depression in HD. Furthermore, the findings of the study are in keeping with the studies that suggest that apathy may start early in the disease. Another interesting finding is that in this group of subjects, there is an association with moderate scores on the cognitive scores (sdmt), again implying that subjects with severe disease as denoted by the extreme catabolic state have evidence of moderate to severe cognitive impairment early on in the disease process, i.e. in the premanifest stage. Interestingly, this was not the case with MMSE (i.e., mmsetotal), adding further evidence that sdmt may be more sensitive at detecting cognitive dysfunction in HD. This has already been demonstrated in the TRACK-HD study [11].

The results in the other category wtCat -2 in premanifest subjects demonstrate slightly different associations. In this case, weight-loss was associated largely with older age (>60 years), again with moderate functional independence impairment, tfcscore 1 and either moderate depression or high apathy scores. It is difficult to establish from these findings whether late onset disease as in this group is associated with more prominent apathy and executive scores.

In the family control group of subjects, the distribution of the age is different which may affect the results in that the highest number was in age group (50–70 years) shown in Fig 2. However, as one would expect, wtCat -2 was associated with severe depression (scores 2, 3) and mild irritability and apathy. On the other hand, extreme weight-loss was associated with older age and moderate functional impairment without depression. This may mean that the weight-loss is attributed to a second pathology. Only in one rule did severe depression come up in this group, interestingly in association with moderate cognitive scores, sdmt = 2.

This work is a preliminary study looking at a novel way of assessing multiple clinical parameters with complex associations with a combination of machine intelligence and clinical domain knowledge. The authors believe, that provided the parameters are selected carefully by a clinical expert and categorized, significant correlations can be better studied compared with the case of adopting classical statistical approach. There are few shortcomings in the study (especially in relation to attribute interaction and related domain knowledge) that would need to be refined before a larger and more extensive study could be undertaken.

## 5. Conclusions and future directions

This paper uses a combination of clinical domain expert knowledge and data analytics algorithms to investigate the non-motor features of Huntington’s disease (HD) that may be highly associated with severe weight-loss, specifically, in the early premanifest/non-motor stage. We transformed a 5 year Enroll-HD dataset into knowledge base (i.e., the categorical form) driven by clinical expertise, which was then processed by the intelligent algorithms to identify the complex behaviors of selected factors. The study performed detailed analysis using well established clustering and class rule mining algorithms to investigate weight-loss associations with multiple non-motor factors in the premanifest stage of HD. Our results demonstrate that certain psychiatric features, namely depression, irritability and apathy as well as cognitive impairment and functional independence were significantly associated with severe weight-loss in the premanifest stage of HD. This is a potentially important finding, implying that weight-loss could be used as a biological marker in the early stages of the disease. These results may also be investigated further in future studies aiming at understanding the dysfunction of neuronal circuits in HD. Furthermore, using this clinical expertise informed analytics approach lends itself to being applicable across later stages of HD, to help investigate whether weight-loss could be used as a biological marker for disease monitoring in therapeutic trials.

## References

[1] Andrews S.C., Domínguez J.F, Mercieca E.C., Karistianis G.N, & Stout J.C. (2015). Cognitive interventions to enhance neural compensation in Huntington’s disease, *Neurodegenerative Disease Management*, 5(2), 155–164. doi: 10.2217/nmt.14.58 25894879

[2] Gregory S., Long J.D., Klöppel S., et al. (2017). Operationalizing compensation over time in neurodegenerative disease. *Brain*, 140(4), 1158–1165, doi: 10.1093/brain/awx022 28334888 PMC5382953

[3] Soloveva M.V., Jamadar S.D., Poudel G., & Georgiou K.N. (2018). A critical review of brain and cognitive reserve in Huntington’s disease, *Neuroscience & Biobehavioral Reviews*, 88, 155–169. doi: 10.1016/j.neubiorev.2018.03.003 29535068

[4] Rao J.A., Harrington D., Durgerian S., et al. (2014). Disruption of response inhibition circuits in prodromal Huntington disease. *Cortex*: *a Journal Devoted to the Study of the Nervous System and Behavior*, 58, 72–85, doi: 10.1016/j.cortex.2014.04.018 24959703 PMC4227536

[5] Kloppel S., Draganski B., Siebner H.R., Tabrizi S.J., Weiller C., & Frackowiak R.S. (2009). Functional compensation of motor function in pre-symptomatic Huntington’s disease. *Brain*, 132 (6), 1624–1632. doi: 10.1093/brain/awp081 19369489 PMC2685920

[6] Malejko K., Weydt P., Süßmuth S.D., Grön G., Landwehrmeyer B.G., & Abler B., B. (2014). Prodromal huntington disease as a model for functional compensation of early neurodegeneration. *PLOS ONE*, 9(12), doi: 10.1371/journal.pone.0114569 25541992 PMC4277279

[7] Georgiou K.N., Poudel G.R., Langmaid R., et al. (2013). Functional and connectivity changes during working memory in Huntington’s disease: 18-month longitudinal data from the IMAGE-HD study, *Brain and Cognition*, 83(1), 80–91. doi: 10.1016/j.bandc.2013.07.004 23938592

[8] Emilia M.G., Natalia G. R., Gabriel P., José L. E., Martín E. C., & Claudia P. (2020). Huntington disease: Advances in the understanding of its mechanisms. *Clinical Parkinsonism & Related Disorders*, 03, 10.1016/j.prdoa.2020.100056. PMC8298812 34316639

[9] David W.W., Aaron S., & Blair R.L. (2011). Development of biomarkers for Huntington’s disease. *The Lancet Neurology*, 10(6), 573–590. doi: 10.1016/S1474-4422(11)70070-9 21601164

[10] Tabrizi S.J., Langbehn D.R., Leavitt B.R., Roos R.A., Durr A., Craufurd D., et al. (2009). TRACK-HD investigators Biological and clinical manifestations of Huntington’s disease in the longitudinal TRACK-HD study: cross-sectional analysis of baseline data. *Lancet Neurol*. 8(9), 791–801. doi: 10.1016/S1474-4422(09)70170-X 19646924 PMC3725974

[11] Tabrizi S.J., Scahill R.I., Durr A., Roos R.A., Leavitt B.R., Jones R., et al. (2011). Biological and clinical changes in premanifest and early stage Huntington’s disease in the TRACK-HD study: The 12-month longitudinal analysis. Lancet Neurol, 10(1), 31–42. doi: 10.1016/S1474-4422(10)70276-3 21130037

[12] Sylvia K., & Aleksey G. K. (2011). Huntington’s disease: From molecular basis to therapeutic advances. *The International Journal of Biochemistry & Cell Biology*, 43(1), 20–24. 21056115 10.1016/j.biocel.2010.10.014

[13] Burg J.M.M., Björkqvist M., & Brundin B. (2009). Beyond the brain: widespread pathology in Huntington’s disease. *Lancet Neurol*, 08, 765–774. doi: 10.1016/S1474-4422(09)70178-4 19608102

[14] David N., & Ruskin J.L., Ross M.K., Tiffany L., Ruiz J.D., & Susan A. M. (2011). A ketogenic diet delays weight-loss and does not impair working memory or motor function in the R6/2 1J mouse model of Huntington’s disease. *Physiology & Behavior*, 103(5), 501–507. doi: 10.1016/j.physbeh.2011.04.001 21501628 PMC3107892

[15] Sanberg P.R., Fibiger H.C., & Mark R.F. (1981). Body weight and dietary factors in Huntington’s disease patients compared with matched controls. *Med J Aust*, 01, 407–09. doi: 10.5694/j.1326-5377.1981.tb135681.x 6454826

[16] Trejo A., Tarrats R.M., Alonso M.E., Boll M.C., Ochoa A., & Velasquez L. (2004). Assessment of the nutrition status of patients with Huntington’s disease. *Nutrition*, 20, 192–196. doi: 10.1016/j.nut.2003.10.007 14962685

[17] Morales L.M., Estevez J., Suarez H., Villalobos R., Chacin B.L., & Bonilla E. (1989) Nutritional evaluation of Huntington disease patients. *Am J Clin Nutr*, 50, 145–50. doi: 10.1093/ajcn/50.1.145 2526577

[18] Djousse L., Knowlton B., Cupples L.A., Marder K., Shoulson I., & Myers R.H. (2002) Weight-loss in early stage of Huntington’s disease. *Neurology*, 59, 1325–1330. doi: 10.1212/01.wnl.0000031791.10922.cf 12427878

[19] Mochel F., Charles P., Seguin F., et al. (2007). Early energy deficit in Huntington disease: identification of a plasma biomarker traceable during disease progression. *PLoS ONE*, 2, e647. doi: 10.1371/journal.pone.0000647 17653274 PMC1919424

[20] Burg J.M., Bacos K., Wood N.I, et al. (2008). Increased metabolism in the R6/2 mouse model of Huntington’s disease. *Neurobiol Dis*, 29, 41–51. doi: 10.1016/j.nbd.2007.07.029 17920283

[21] Goodman A.O., Murgatroyd P.R., Medina-Gomez G., et al. (2008). The metabolic profile of early Huntington’s disease: a combined human and transgenic mouse study. *Exp Neurol*, 210, 691–98. doi: 10.1016/j.expneurol.2007.12.026 18284928

[22] Myers R.H., Sax D.S., Koroshetz W.J., et al. (1991). Factors associated with slow progression in Huntington’s disease. *Arch Neurol*, 48, 800–804. doi: 10.1001/archneur.1991.00530200036015 1832854

[23] Aziz N.A., Burg J.M.M., Landwehrmeyer G.B., Brundin P., & Stijnen T. (2008). Weight-loss in Huntington disease increases with higher CAG repeat number. *Neurology*, 71(19), 1506–1513. doi: 10.1212/01.wnl.0000334276.09729.0e 18981372

[24] Petersén A., & Björkqvist M. (2006). Hypothalamic–endocrine aspects in Huntington’s disease. *European Journal of Neuroscience*. doi: 10.1111/j.1460-9568.2006.04985.x 16925587

[25] Cheong R.Y., Gabery S., & Petersén Å. (2019). The Role of Hypothalamic Pathology for Non-Motor Features of Huntington’s Disease. *J Huntingtons Dis*, 8(4), 375–391. doi: 10.3233/JHD-190372 31594240 PMC6839491

[26] Angela R., Diederick S., Sarah S., Josh K., Anders D., Jody G., et al. (2011). Evaluating imaging biomarkers for neurodegeneration in pre-symptomatic Huntington’s disease using machine learning techniques. *NeuroImage*, 56(2), 788–796, doi: 10.1016/j.neuroimage.2010.04.273 20451620

[27] Graziella O., William P., Andre F.M., Giuseppe S., & Andrea M. (2012). Using Support Vector Machine to identify imaging biomarkers of neurological and psychiatric disease: A critical review. *Neuroscience & Biobehavioral Reviews*, 36(4), 1140–1152. 22305994 10.1016/j.neubiorev.2012.01.004

[28] Clara G., Alberto L., Saul M., Jesus P., Jaime K., Nadia R., et al. (2019). Specific patterns of brain alterations underlie distinct clinical profiles in Huntington’s disease. *NeuroImage*: *Clinical*, 23, 101900. 10.1016/j.nicl.2019.101900 PMC6606833 31255947

[29] PDS4, (2018). Enroll-HD: A worldwide observational study for Huntington’s disease, families. A CHDI Foundation Project, Retrieved from: https://www.enroll-hd.org/enrollhd_documents/2018-10-R1/Enroll-HD-DataDictionary-2018-10-R1.pdf.

[30] Vasan D., Alazab M., Wassan S., Safaei B., Zheng Q. (2020). Image-Based malware classification using ensemble of CNN architectures (IMCEC). *Computers & Security*, 92, 10.1016/j.cose.2020.101748.

[31] Bhattacharya S., Kumar P., Maddikunta R. et al. (2021). Deep learning and medical image processing for coronavirus (COVID-19) pandemic: A survey. *Sustainable Cities and Society*, 65, doi: 10.1016/j.scs.2020.102589 33169099 PMC7642729

[32] Gadekallu T.R., Alazab M., Kaluri R. et al. (2021). Hand gesture classification using a novel CNN-crow search algorithm. *Complex Intell*. *Syst*, 10.1007/s40747-021-00324-x.

[33] Khan W., Crocket K., Oshea J., Hussain A., Khan B. (2021). Deception in the Eyes of Deceiver: A Computer Vision and Machine Learning Based Automated Deception Detection. *Expert Systems with Applications*, Elsevier, 169, 10.1016/j.eswa.2020.114341

[34] Khan W., Hussain A., Kuru K., Al-askar H. (2020) Pupil Localisation and Eye Centre Estimation using Machine Learning and Computer Vision. *Sensors*, 20(13), doi: 10.3390/s20133785 32640589 PMC7374404

[35] Kutia S., Chauhdary S.H., Iwendi C., Liu L., Yong W., Bashir A.K. (2019). Socio-Technological Factors Affecting User’s Adoption of eHealth Functionalities: A Case Study of China and Ukraine eHealth Systems. IEEE Access, 07, 90777–90788, doi: 10.1109/ACCESS.2019.2924584

[36] Iwendi C., Moqurrab S.A., Anjum A., Khan S., Mohan S., Srivastava G. (2020). N-Sanitization: A semantic privacy-preserving framework for unstructured medical datasets, *Computer Communications*, 161, 160–171, 10.1016/j.comcom.2020.07.032.

[37] Khan W., Hussain A., Sohail K., Jumailey M., Nawaz R., Liatsis P. (2021). Analysing the Impact of Global Demographic Characteristics over the COVID-19 Spread Using Class Rule Mining and Pattern Matching. *Royal Society Open Science*, 08, 01–19, doi: 10.1098/rsos.201823 33614100 PMC7890495

[38] Kohonen T. (1990). The self-organizing map. Proceedings of the IEEE, 78(09), 1464–1480, doi: 10.1109/5.58325

[39] Agrawal A., Mannila H., Srikant R., Toivonen H., & Verkamo A. (1996). Fast Discovery of Association Rules. In: Fayyad et al. (1996), 307–328.

[40] Naulaerts S., Meysman P., Bittremieux W., et al. (2015) A primer to frequent itemset mining for bioinformatics. *Brief Bioinform*, 16, 216–231. doi: 10.1093/bib/bbt074 24162173 PMC4364064

[41] Nahar J., Imam T., Tickle K., & Chen Y.P. (2013). Association rule mining to detect factors which contribute to heart disease in males and females. *Expert Syst*. *Appl*. *Elsevier*, 40, 1086–1093.

[42] Ordonez C. (2006). Association rule discovery with the train and test approach for heart disease prediction. *IEEE Trans*. *Inf*. *Technol*. *Biomed*. 10(02), 334–343, doi: 10.1109/titb.2006.864475 16617622

[43] Luna J., Romero C., Romero J., & Ventura S. (2015). An evolutionary algorithm for the discovery of rare class association rules in learning management systems. *Appl*. *Intell*, 42, 501–513.

[44] Ali S., Mehmood F., Darren D., et al., (2019). An Adaptive Multi-Robot Therapy for Improving Joint Attention and Imitation of ASD Children. *IEEE Access*, 07, 81808–81825, doi: 10.1109/ACCESS.2019.2923678

[45] Gonçalves E.C., Mendes I.M.B., & Plastino A. (2004) Mining Exceptions in Databases. In: Webb G.I., Yu X (eds) AI 2004: *Advances in Artificial Intelligence*. AI 2004. Lecture Notes in Computer Science, 3339, Springer, Berlin, Heidelberg.

[46] Michael Hahsler A Probabilistic Comparison of Commonly Used Interest Measures for Association Rules, 2015, URL: http://michael.hahsler.net/research/association_rules/measures.html

[47] Yingquan, W., & Tomohiro, M. (2017). Association Rule Mining with Data Item including Independency based on Enhanced Confidence Factor, Proceedings of the International Multi Conference of Engineers and Computer Scientists, 01, IMECS, Hong Kong.

[48] Bayardo R., Agrawal R., & Gunopulos D. (2000). Constraint-based rule mining in large, dense databases. *Data Mining and Knowledge Discovery*, 04(2/3), 217–240.

[49] Vadlamudi China Venkaiah, Deepika Vadlamudi Sesha Phani, Chapter 4—Mathematical Essentials, Handbook of Statistics, Elsevier, Volume 38,2018,Pages 53–73,ISBN 9780444640420, 10.1016/bs.host.2018.07.008.

[50] Xin D., Terence Y.C.P., & Anthony J.H. (2013). A Tale of Two Maladies? Pathogenesis of Depression with and without the Huntington’s Disease Gene Mutation. *Front Neurol*, 4(81). 10.3389/fneur.2013.00081 PMC3705171 23847583

[51] Vassos E., Panas M., Kladi A., & Vassilopoulos D. (2008). Effect of CAG repeat length on psychiatric disorders in Huntington’s disease. *J*. *Psychiatr*. *Res*. 42(07), 544–549, doi: 10.1016/j.jpsychires.2007.05.008 17610899

[52] Julien L., Thompson C., Wild S., et al. (2007). Psychiatric disorders in preclinical Huntington’s disease. *J Neurol Neurosurg Psychiatry*, 78, 939–943. doi: 10.1136/jnnp.2006.103309 17178819 PMC2117854

[53] Epping E.A., Kim J.I., Craufurd D., et al. (2016). PREDICT‐HD Investigators and Coordinators of the Huntington Study Group. Longitudinal psychiatric symptoms in prodromal Huntington’s disease: a decade of data. *Am J Psychiatry*, 173–184. doi: 10.1176/appi.ajp.2015.14121551 26472629 PMC5465431

[54] van-Duijn E., Reedeker N., Giltay E.J., Eindhoven D., Roos R.A., & van-der-Mast R.C. (2014). Course of irritability, depression and apathy in Huntington’s disease in relation to motor symptoms during a two‐year follow‐up period. *Neurodegener Dis*, 9–16. doi: 10.1159/000343210 23948661

[55] Saul M., Jesus P., Erik D., Ramon F., Mar C., Javier P., et al. (2016). Neuropsychiatric Symptoms Are Very Common in Premanifest and Early Stage Huntington’s Disease. *Parkinsonism Relat Disord*, 25, 58–64, doi: 10.1016/j.parkreldis.2016.02.008 26898966

